# Melanoma Metastasis to the Heart: “Myectomy” With Immunotherapy

**DOI:** 10.1016/j.jaccas.2024.102862

**Published:** 2025-01-02

**Authors:** Basilio Angulo-Lara, Fernando Domínguez

**Affiliations:** a Department of Cardiology, Puerta de Hierro University Hospital, Madrid, Spain; b Biomedical Research Network Center for Cardiovascular Research (CIBERCV), Madrid, Spain

**Keywords:** cardiac magnetic resonance, cardiac mass, immunotherapy, metastatic melanoma

## Abstract

A 44-year-old man presented to the emergency department with high-risk cardiogenic syncope. Investigations revealed a cardiac mass, corresponding to metastatic melanoma. However, the primary tumor was not found. Thanks to the histologic study, targeted immunotherapy with nivolumab and ipilimumab was started and showed an excellent response in follow-up.

## Graphical Abstract

**Figure:**
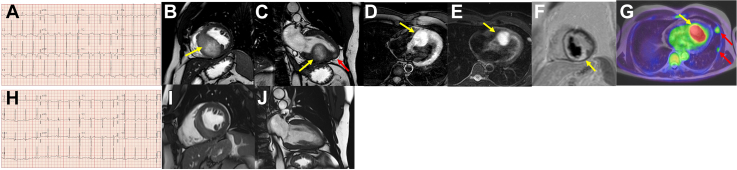

**Take-Home Messages**

• This case highlights the importance of advanced imaging techniques and endomyocardial biopsy when diagnosing metastatic melanoma involving the heart, especially when the primary tumor is not identifiable.
• Although surgery was not possible given the tumor’s location, this case demonstrates the potential of medical treatments in addressing a cardiac tumor.

A 44-year-old man with no medical history of interest presented to the emergency department after a single episode of syncope at rest, without prodrome and with complete recovery within a few minutes. He was asymptomatic for chest pain, dyspnea, or palpitations. On examination, his vital signs were normal, with blood pressure of 130/85 mm Hg, heart rate of 70 beats/min, and 97% oxygen saturation on room air. Cardiopulmonary auscultation was unremarkable, and there were no signs of focal neurologic deficits. The electrocardiogram (ECG) showed sinus rhythm with deep negative T waves in the inferior and precordial leads (V_4_-V_6_) (Figure 1A). A transthoracic echocardiogram showed a bulky mass occupying the interventricular septum, and this mass (5 cm × 6 cm)was confirmed by a cardiac magnetic resonance (CMR) imaging study (Figures 1B and 1C, Videos 1 and 2). CMR confirmed the presence of a septal mass, with hypointense signal in T1, prolonged relaxation time in T2, and mild peripheral enhancement after gadolinium administration (Figures 1D to 1F). In addition, it showed an apical satellite nodule and pulmonary nodules in the left hemithorax that were suggestive of metastases. Positron emission tomography with computed tomography was performed, without a hint of an evident primary tumor (Figure 1G). The histologic study of a pulmonary nodule revealed metastatic melanoma with a *BRAF* sequence variant. Several suspicious nevi were biopsied, but the primary tumor was not found. The Oncology Department started immunotherapy with nivolumab and ipilimumab, with an excellent cardiac response. After 12 months of follow-up, the ECG normalized, and the cardiac mass was drastically reduced in size (Figures 1H to 1J, Video 3). He is in good general condition and is active on a daily basis, with no new syncopal episodes.

**Figure 1:**
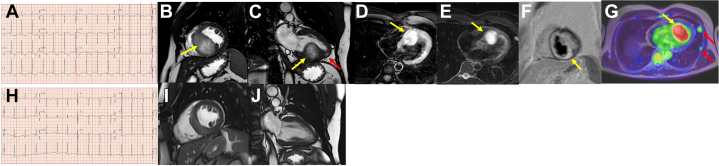
**Melanoma Metastasis to the Heart** (A) First electrocardiogram. Sinus rhythm with signs of left ventricular hypertrophy and negative T waves on the inferolateral leads. (B and C) First cardiac magnetic resonance, short-axis and 2-chamber view cine magnetic resonance, showing an interventricular and apical cardiac mass with hypersignal (yellow arrows and red arrows, respectively). (D) Cardiac magnetic resonance, native T1 sequence, showing a septal mass (yellow arrow) with high intensity signal (longer T1 relaxation time). (E) Cardiac magnetic resonance, T2 sequence, showing a septal mass (yellow arrow) with prolonged relaxation time. (F) Cardiac magnetic resonance, showing a septal mass with mild peripheral enhancement after gadolinium administration (yellow arrow). (G) Positron emission tomography with computed tomography. A cardiac mass (yellow arrow) showing behavior suggestive of metabolic aggressiveness (hyperenhancement with a central hypometabolic area compatible with central necrosis). Multiple pulmonary nodules suggestive of malignancy (red arrows) are present. (H) Second electrocardiogram. Normalization of the repolarization anomalies. (I and J) Second cardiac magnetic resonance, short-axis and 2-chamber view cine magnetic resonance, where we can see a reduction in cardiac mass size.

Metastatic involvement of the heart is 20 times more frequent than primary tumors, which are very rare (between 0.7% and 3.5% in autopsies of the general population).^1^ Melanomas are particularly predisposed to metastasize to the heart. Survival of metastatic melanoma is slightly more than 5 years on average.^1^ In our case, in the absence of a primary tumor, biopsy was key to clarifying its histologic origin, which is very relevant in deciding the most accurate type of chemotherapy.^2^^,^^3^ Because of the advanced disease and the location of the mass inside the interventricular septum, the patient could not benefit from surgical treatment.

This case shows a great response to targeted immunotherapy treatment and proves how a medical treatment without surgery can drastically reduce a malignant's tumor mass and consequently normalize the ECG.

## Funding Support and Author Disclosures

The authors have reported that they have no relationships relevant to the contents of this paper to disclose.

## Appendix

Video 1 Transthoracic Echocardiography, 4-Chamber View, Showing an Interventricular Cardiac Mass

Video 2 Transthoracic Echocardiography, Doppler Long-Axis View, Showing an Interventricular Cardiac Mass The interior of the mass is anechoic and shows Doppler flow, The interior of the mass is anechoic and shows Doppler flow, suggesting vascularization of the mass

Video 3 Transthoracic Echocardiography, 4-Chamber View, After 12 Months of Follow-Up, Showing Drastic Reduction of the Cardiac Mass
